# Proton Exchange Membrane (PEM) Fuel Cells with Platinum Group Metal (PGM)-Free Cathode

**DOI:** 10.1007/s42154-021-00146-0

**Published:** 2021-04-28

**Authors:** Lei Du, Gaixia Zhang, Shuhui Sun

**Affiliations:** 1 Institut National de la Recherche Scientifique (INRS)-Énergie Matériaux et Télécommunications, Varennes, QC J3X 1S2 Canada; 2 School of Chemistry and Chemical Engineering, Harbin Institute of Technology, Harbin, 150001 China

**Keywords:** PEM fuel cells, Cathode, PGM-free catalysts, Performance, Stability

## Abstract

Proton exchange membrane (PEM) fuel cells have gained increasing interest from academia and industry, due to its remarkable advantages including high efficiency, high energy density, high power density, and fast refueling, also because of the urgent demand for clean and renewable energy. One of the biggest challenges for PEM fuel cell technology is the high cost, attributed to the use of precious platinum group metals (PGM), e.g., Pt, particularly at cathodes where sluggish oxygen reduction reaction takes place. Two primary ways have been paved to address this cost challenge: one named low-loading PGM-based catalysts and another one is non-precious metal-based or PGM-free catalysts. Particularly for the PGM-free catalysts, tremendous efforts have been made to improve the performance and durability—milestones have been achieved in the corresponding PEM fuel cells. Even though the current status is still far from meeting the expectations. More efforts are thus required to further research and develop the desired PGM-free catalysts for cathodes in PEM fuel cells. Herein, this paper discusses the most recent progress of PGM-free catalysts and their applications in the practical membrane electrolyte assembly and PEM fuel cells. The most promising directions for future research and development are pointed out in terms of enhancing the intrinsic activity, reducing the degradation, as well as the study at the level of fuel cell stacks.

## Introduction

Proton exchange membrane (PEM) fuel cells, particularly the ones using hydrogen at the anode and oxygen or air at the cathode, namely H_2_ PEM fuel cells, can generate electricity without carbon emission. PEM fuel cells work at low temperatures (e.g., 60–80 ℃), have high efficiency, high power, and energy density, and can be refueled fast. In this regard, PEM fuel cells are widely regarded as one of the most promising power sources for electric vehicles (EVs).

**Fig. 1:**
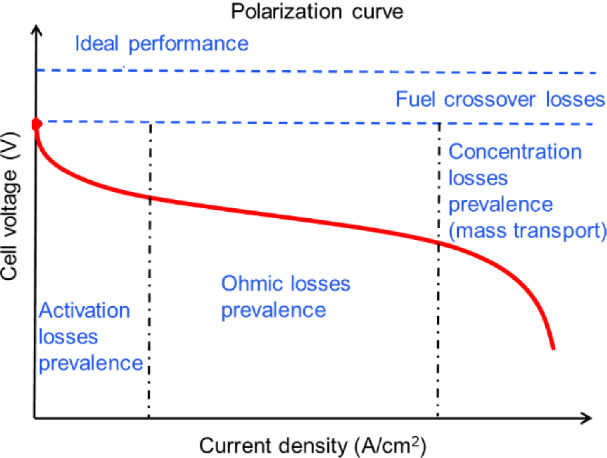
The typical polarization curve for PEM fuel cells [1]

For PEM fuel cells, a typical polarization curve is shown in Fig. 1 [1]. The theoretical output voltage of PEM fuel cells is 1.23 V if the hydrogen and oxygen are fed at the anode and cathode, respectively. However, in practice, the polarization usually takes place, i.e., overpotentials are needed. The overall polarization is caused by fuel crossover, kinetics polarization at low current density range, ohmic polarization at medium current density range, and concentration polarization at high current density range. The fuel crossover is primarily determined by the membrane; the other three polarizations (i.e., kinetics, ohmic, and concentration) are all closely relevant to the catalysts. That is to say, the catalysts should catalyze the reactions with high kinetics so that the kinetics polarization is minimized, i.e., low activation losses in Fig. 1. The catalysts are also required to have high electronic conductivity and desired porous structures (channels for mass transfer) to suppress the ohmic and concentration losses. Therefore, catalysts are a key component of PEM fuel cells.

The most widely applied catalysts are based on PGM, e.g., Pt, in PEM fuel cells. As estimated by the U.S. Department of Energy, the platinum group metal (PGM) catalysts occupy over 40% of the total fuel cell stack cost at large-scale production (assuming 500,000 systems per year) [2]. The use of PGM catalysts thus leads to the high cost and hinders the deployments of fuel cells in EVs.

The majority of PGM catalysts are consumed at cathode because of the much more sluggish kinetics of cathodic oxygen reduction reaction (ORR) than the coupled anodic hydrogen oxidation reaction. Thus, decreasing the usage of PGM catalysts and even replacing them with PGM-free alternatives for cathode are two promising strategies for PEM fuel cell applications. This paper mainly focuses on the PGM-free catalysts for the cathode of PEM fuel cells. (Without specific emphasis, the discussed catalysts in the following contents are referring to the cathode catalysts.) Section 2 presents the main challenges for PGM-free catalysts in terms of performance and durability/stability; Sects. 3 and 4 demonstrate the recent progress of high-performance and highly durable/stable PGM-free catalysts; in Sect. 5, the gaps between the current status and the future perspectives are identified. Several promising strategies to address the challenges are pointed out as well.

## Main Challenges for PGM-Free Catalysts in PEM Fuel Cells

**Fig. 2:**
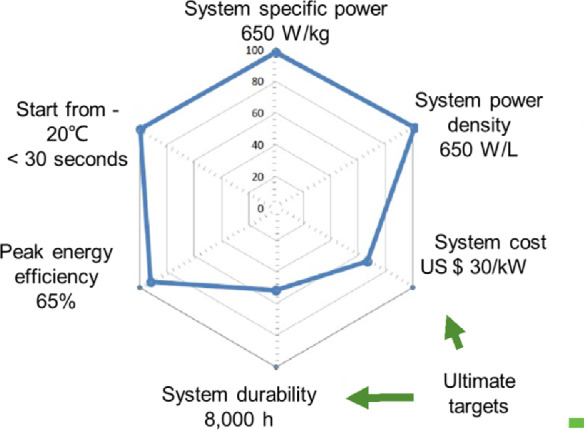
The greatest challenges for fuel cell applications [2]

PEM fuel cells with PGM catalysts have been applied in EVs. For example, the Toyota Mirai using PtCo alloying catalysts at cathode has been successfully launched in the market. However, current PEM fuel cells are not quite satisfied to meet all the requirements. The most challenging aspects are cost and durability, as shown in Fig. 2.

The performance of PEM fuel cells using PGM-free cathode has been significantly improved in the past decades, approaching the proposed targets, while the lifetime still needs great effort. The following sections will focus on the performance and durability/stability challenges for the PGM-free cathode.

### Performance Challenge for PEM Fuel Cells with PGM-Free Catalysts

**Fig. 3:**
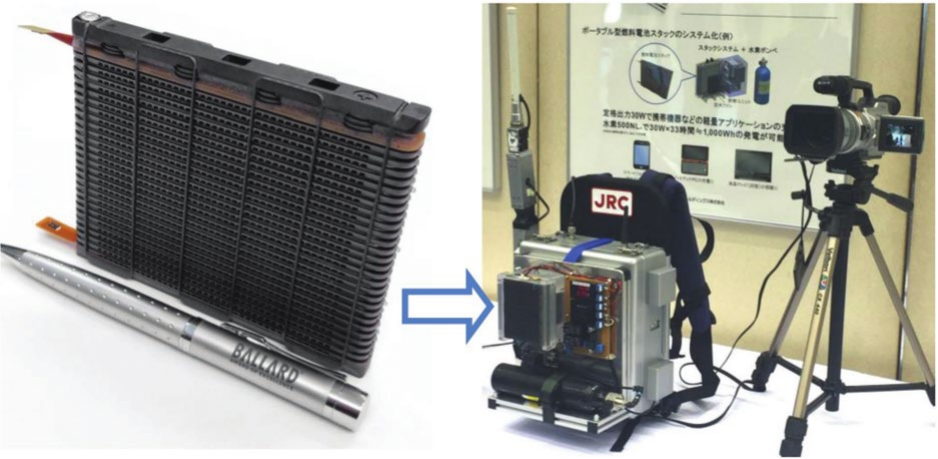
FCgen-micro fuel cell that can power the emergency power/wifi backpack [3]

The world’s first PEM fuel cell prototype (named FCgenmicro) was released by Ballard Power Systems using the PGM-free catalysts at the cathode. This micro-PEM fuel cell (only 146 g in weight) can output a rated power of 30 W at 2.4 A, which can power portable devices like the one developed by Japan Radio Co. (Fig. 3) [3]. This milestone inspires the research and development of PGM-free catalysts for PEM fuel cells although the performance still does not meet the EV requirements.

**Fig. 4:**
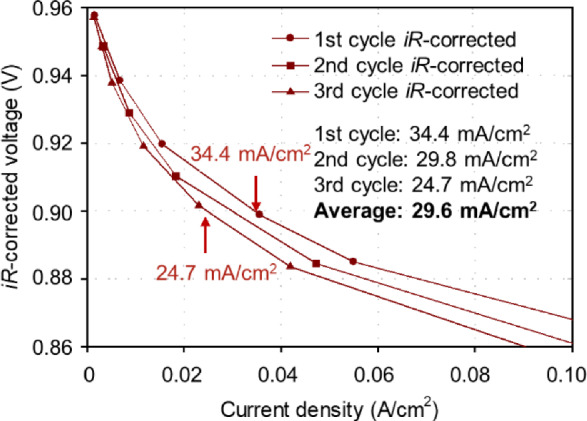
The PEM fuel cell performance of CM-PANI-Fe-C(Zn) cathode catalyst [6]

To promote the application of PGM-free catalysts in real-world EVs, the corresponding performance targets have been proposed for PEM fuel cells. It should be emphasized that compared with the widely used half-cell tests, i.e., using the rotating (ring) disk electrode, the evaluations in the membrane electrode assembly (MEA) are closer to the real-world fuel cells. Thus, the targets are all proposed based on MEA tests. The performance target for MEA with PGM-free catalysts is over 44 mA/cm^2^ at 900 mV_IR-free_ in the H_2_/O_2_ fuel cell at 100 kPa partial pressure of O_2_ and cell temperature 80 °C [4, 5], which is equivalent to the PGM-based catalyst target: 440 mA/mg_Pt_ × 0.1 mg_Pt_/cm^2^. However, the most state-of-the-art current density at 900 mV_IR-free_ is still lower than 40 mA/cm^2^, as shown in Fig. 4, indicating that efforts are still needed to achieve the desired PGM-free catalysts for PEM fuel cells.

### Durability/Stability Challenge for PEM Fuel Cells with PGM-Free Catalysts

In addition to the performance, lifetime is another grand challenge for PEM fuel cells with PGM-free catalysts. As shown in Fig. 2, the targets 5000 h by 2025 to the ultimate 8000 h beyond 2030 for the light-duty EVs. The above-mentioned FCgen-micro fuel cell can work for more than 500 h, which, however, is still far from meeting the requirements of thousands of hours.

The durability and stability of catalysts significantly determine the lifetime of the MEA and PEM fuel cells. It is noticed that in the past decades, the activity of PGM-free catalysts has been remarkably improved, represented by the breakthroughs achieved by the Dodelet group at Institut National de la Recherche Scientifique (INRS) [7, 8] and Zelenay group at Los Alamos National Laboratory (LANL) [9–11]. However, the high-performance PGM-free catalysts usually suffer from rapid degradation under ORR environments [12, 13].

**Fig. 5:**
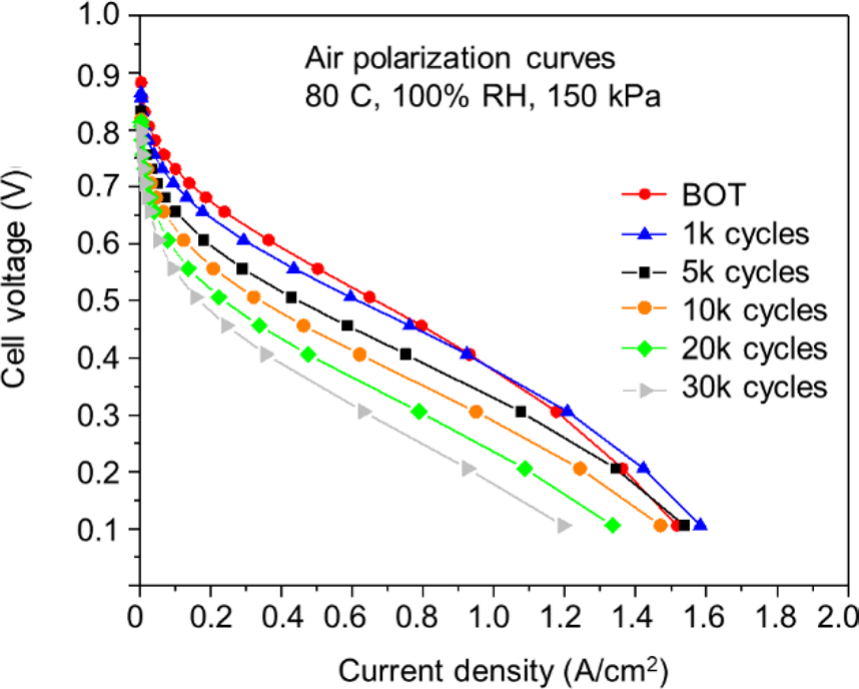
Polarization curves after cycles using square-wave AST protocol in the air [16]

To understand and evaluate the degradation of PGM-free catalysts, “durability” and “stability”, which are estimated by the performance loss after voltage cycling and potentiostatic/galvanostatic tests, respectively, have been widely investigated [14]. Learning from the accelerated stress test protocol (AST, square wave between 0.6 and 0.95 V_3s) for PGM catalyst cases [15], the counterpart AST protocol using square wave cycles between 0.6 V_3s and OCV (or a specific voltage value)_3s has been applied in research to evaluate the durability of PGM-free catalysts in PEM fuel cells [6, 16]. As shown in Fig. 5, the polarization curves at the beginning of the test and a specific number (1 k, 5 k, 10 k, 20 k, and 30 k) of 0.6–0.95 V cycle in the air are recorded in the PEM fuel cell MEA at 80 ℃ under 150 kPa absolute pressure and 100% RH. It can be seen that the durability of PGM-free catalysts still needs improvement.

**Fig. 6:**
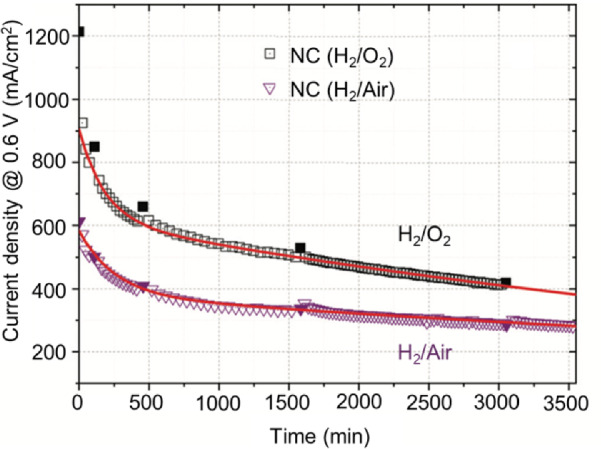
Stability behavior at 0.6 V of Fe-N-C in H_2_/O_2_ and in H_2_/air PEM fuel cells, as well as the superimposed curves fitted according to the INRS model [20]

Other than the durability test using voltage cycling, the stability tests using potentiostatic/galvanostatic protocols, i.e., fixed voltage and current, particularly the former, were more widely used in PGM-free catalyst investigations in laboratories [17–19]. In 2011, the INRS team developed a highly active and high-performing Fe–N–C catalyst, which was able to provide a maximum power density up to 0.91 W/cm^2^ at the cathode of a H_2_/O_2_ PEM fuel cell [7]. This catalyst, however, is not stable. As shown in Fig. 6, for an MEA with this catalyst at the cathode, more than 50% of initial performance can be lost after the first 50-h operation at 0.6 V [20]. This fast degradation phenomenon, in fact, has been widely observed for most PGM-free catalysts, especially the high-performance ones. In the past ten years, the INRS team has been continuously focusing on the stability problem of PEM fuel cells using this high-performance catalyst as a representative [20–23]. So far, the team has been able to interpret the cause of the fast decay and to attribute it to the demetallation of FeN_4_ catalytic sites located in the micropores. Great effort is still needed to identify the slow decay and to see whether it is also attributable to the demetallation of the FeN_4_ sites or another cause, like a chemical attack by H_2_O_2_ (produced electrochemically by ORR and eventually followed by the attack of the catalyst by HO radicals generated by the Fenton reaction).

From the above discussions, it can be seen that the performance and, particularly, the insufficient durability/stability are still the big challenges in PEM fuel cells. Till today, the community has been making great efforts to push the PGM-free PEM fuel cells forward.

## Recent Progress of High-Performance PGM-Free Catalysts

PGM-free catalysts for ORR have been developing for more than fifty years. So far, different types of PGM-free ORR catalysts have been proposed and investigated. For example, some non-carbon materials such as oxides [24, 25] and metal (oxy) nitrides [26, 27] have been used as ORR catalysts, which, however, suffer from low conductivity. Alternatively, carbon-based materials are more promising in practical applications. Metal-free heteroatom doped carbon has been intensively investigated [28]. Even though its intrinsic ORR activity is still lower than the widely known M–N–C materials within MN*x* active moieties [29]. The most promising ORR catalyst is the M–N–C type material. Since this paper targets the practical applications of PGM-free ORR catalyst in the real-world MEA and fuel cells, it mainly discusses the M–N–C catalysts. In the early twenty-first century, several breakthroughs were made toward M–N–C catalysts (Fe–N–C, to be specific) by the INRS team [7, 8] and Los Alamos team [9–11]. Only then did the PGM-free catalysts come into people’s eyes as a promising alternative to replace the widely used PGM catalysts. In the past years, the performance of these PGM-free catalysts has been significantly improved, especially the Fe-based materials. This inspiring progress benefits from the fast understanding of active moieties for PGM-free catalysts as well as the emerging concepts in material science to improve the catalysts’ performance [30].

### Understanding Active Moieties

For PGM-free catalysts, the composite Fe–N–C catalyst including Fe, N, and C is the state-of-the-art catalyst due to its superior performance. The Fe–N–C catalyst is usually synthesized by pyrolyzing the mixed precursors including Fe, N, and C at high temperatures. The high-temperature pyrolysis leads to complicated compositions in final Fe–N–C catalysts. The possible moieties, e.g., FeN*x* and CN*x*, are all involved. Therefore, separating the contributions of different possible active moieties is urgent.

**Fig. 7:**
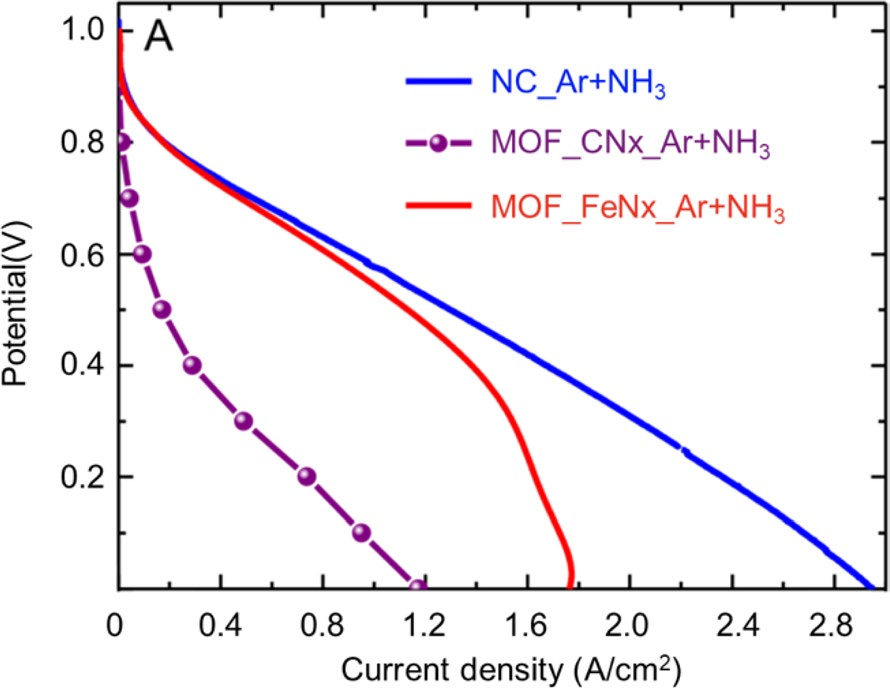
Polarization curve of the Fe-N-C catalyst; the contributions of FeN_*x*_ and CN_*x*_ moieties to the current density at different voltages [29]

The strategy was developed to separate the contributions of FeN*x* and CN*x* moieties to the PEM fuel cell performance [29]. By controlling the Fe concentration in a series of Fe–N–C catalysts and evaluating these catalysts in PEM fuel cells, a linear relationship between log (current density) and log (Fe concentration) can be obtained at a specific cell voltage. Reasonably, by extrapolating the line to the current density point where Fe concentration is 50 ppm (the contribution of FeN*x* can be ignored at such a low concentration), the contribution of CN*x* can be extracted at this voltage. Similarly, a set of current vs. voltage can be obtained to plot the polarization curve of CN*x*. By subtracting CN*x* contribution from the total polarization curve of Fe–N–C catalyst, the polarization curve of FeN*x* is achieved. As shown in Fig. 7, the polarization curves of Fe–N–C catalyst (denoted as NC_Ar + NH_3_), FeN*x* (denoted as MOF_FeN*x*_Ar + NH_3_), and CN*x* (MOF_CN*x*_Ar + NH_3_) indicate that the FeN*x* moiety primarily contributes to the ORR in this Fe–N–C catalyst. In particular, at the high voltage range, the FeN*x* contributes much more than CN*x*, while at the low voltage range, the contribution of CN*x* cannot be ignored anymore.

The configurations of FeN*x* moieties have been investigated. However, the details are still not quite clear in this field. For example, the mainstream believes that FeN*x* moiety with the coordination number of *x* = 4, i.e., FeN_4_, is the real active site; however, it should not be ignored that the coordination number of x in FeN*x* has been also reported as 1–6 [31–35]. Even for the FeN_4_ sites, the detailed structures could be quite different, e.g., FeN_4_ versus FeN_2+2_ [22, 23]; the around carbon matrix could also be significantly involved in the active moieties [36]. Even so, synthesizing atomically dispersed FeN*x* moieties and exposing as much as possible FeN*x* sites (i.e., high active site density) are both beneficial for improving the performance of Fe–N–C catalysts [37].

### Emerging Concepts in Improving the Performance of PGM-free Catalysts

As discussed above, atomically dispersed Fe–N–C catalysts are attractive. Some emerging concepts have been proposed to synthesize advanced Fe–N–C catalysts. Besides, the new M–N–C catalysts beyond Fe–N–C, such as Co–N–C and Mn–N–C, are also proposed.

#### Advanced Fe–N–C Catalysts

To control the atomically dispersed Fe–N–C catalysts, the metal-organic framework (MOF) materials are promising as precursors due to the well-defined metal-nitrogen coordination structures in MOF networks [38]. In 2011, the INRS team used MOF (ZIF-8, to be specific) as the precursor and developed a high-performance Fe–N–C catalyst. Following this work, the MOF has been intensively investigated to synthesize high-performance Fe–N–C catalysts. For example, the size of Fe-doped ZIF-8 precursors could be tuned from 20 to 1000 nm without changing the chemical properties. In this way, the active site density of the derived catalysts was well-tuned, among which the 50 nm Fe–N–C catalyst demonstrated the best intrinsic ORR activity [39]. The usage of Fe in the precursor is important to obtain atomically dispersed FeN*x* moieties: high Fe concentration leads to large, inactive Fe particles and species; low Fe concentration leads to low active site density. Recently, by precisely controlling the doped Fe content in ZIF precursors, the Fe–N–C catalyst exclusively containing atomically dispersed FeN*x* moieties is achieved. The corresponding PEM fuel cell tests indicate 44 mA/cm^2^ at 0.87 V, approaching the target (0.9 V) [40].

**Fig. 8:**
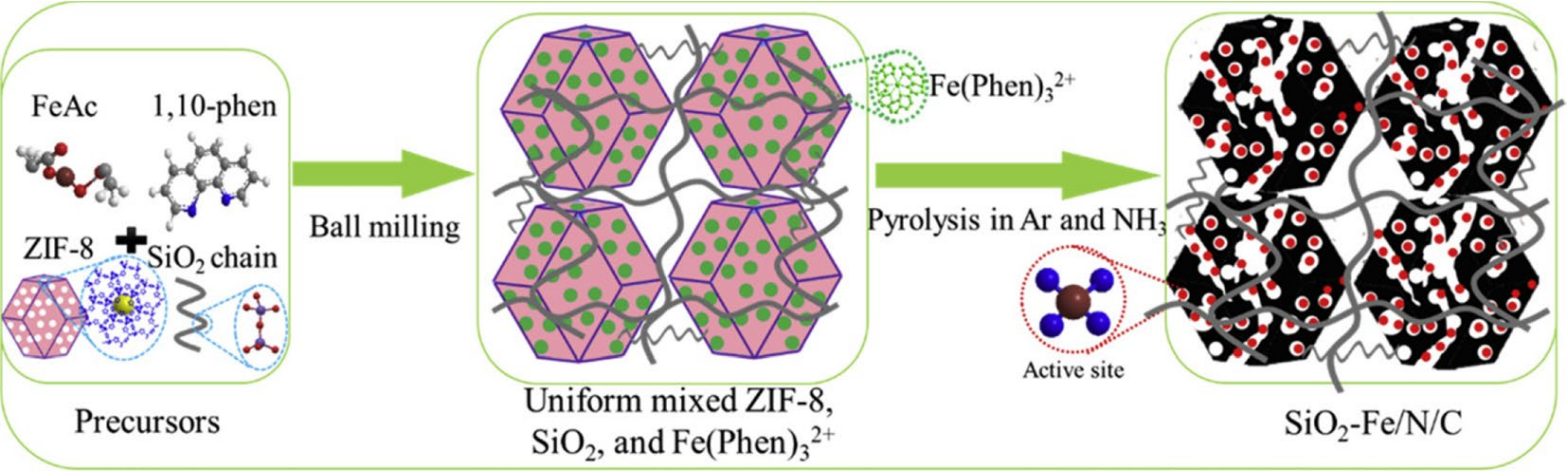
The illustration for the synthetic method for silica mediated Fe-N-C catalyst [42]

In addition to the active moieties themselves, the porous structure is important for high-performance ORR catalysts because the porous structure determines the mass transportation of reactants and products [38, 41]. Following the INRS Fe–N–C catalyst in Ref. [7], the INRS group employed silica to generate abundant channels for mass transfer (Fig. 8) [42]. In high voltage range, e.g., higher than 0.7 V, the fuel cell performances are similar for Fe–N–C catalysts with and without silica. Particularly, at low voltages, the presence of silica significantly enhances the fuel cell performance. For example, at 0.4 V where the mass transport controls performance in H_2_/air fuel cells, the current density of MEA with silica achieves around 780 mA/cm^2^, much higher than the pristine Fe–N–C MEA (620 mA/cm^2^).

**Fig. 9:**
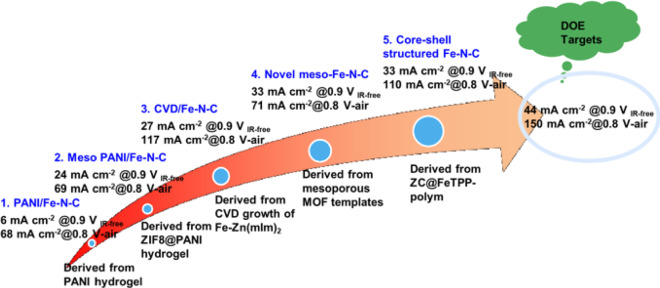
The increasing performance of typical mesoporous Fe-N-C catalysts using template methods [44]

Since the silica is not active for ORR, developing Fe–N–C catalysts with mesoporous structures is promising. As shown in Fig. 9, the performance of mesoporous Fe–N–C catalysts gradually increases and approaches the targets. The silica can also be used as the sacrifice hard template to synthesize Fe–N–C catalysts with mesoporous structure [43]. It should be also emphasized that the reasonably decreased wettability in porous structures accelerates the mass transportation through remitting flooding issue. For example, the introduced hydrophobicity by silica in catalysts was proved beneficial for oxygen transfer [42].

#### Beyond Fe–N–C Catalysts

**Fig. 10:**
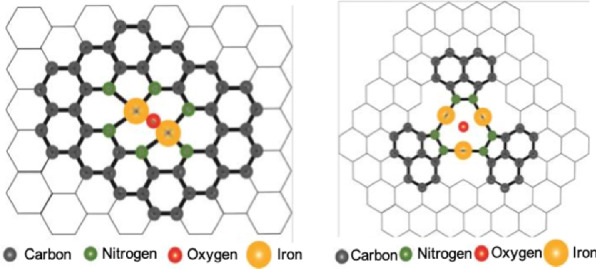
The illustration of catalyst models with multiple Fe centers [47]

To improve the intrinsic ORR activity of Fe–N–C catalysts, novel concepts beyond Fe–N–C catalysts have been proposed. For example, the binary FeM–N–C catalysts have been developed, such as FeCo–N–C [45, 46], FeMn–N–C catalysts [43]. In addition to the FeM–N–C catalysts with bimetallic centers (different metals), an active moiety with more than one Fe center (Fig. 10) is also proposed by using the surface deposition method (chemical vapor deposition) to bypass the widely used pyrolysis methods [47].

**Fig. 11:**
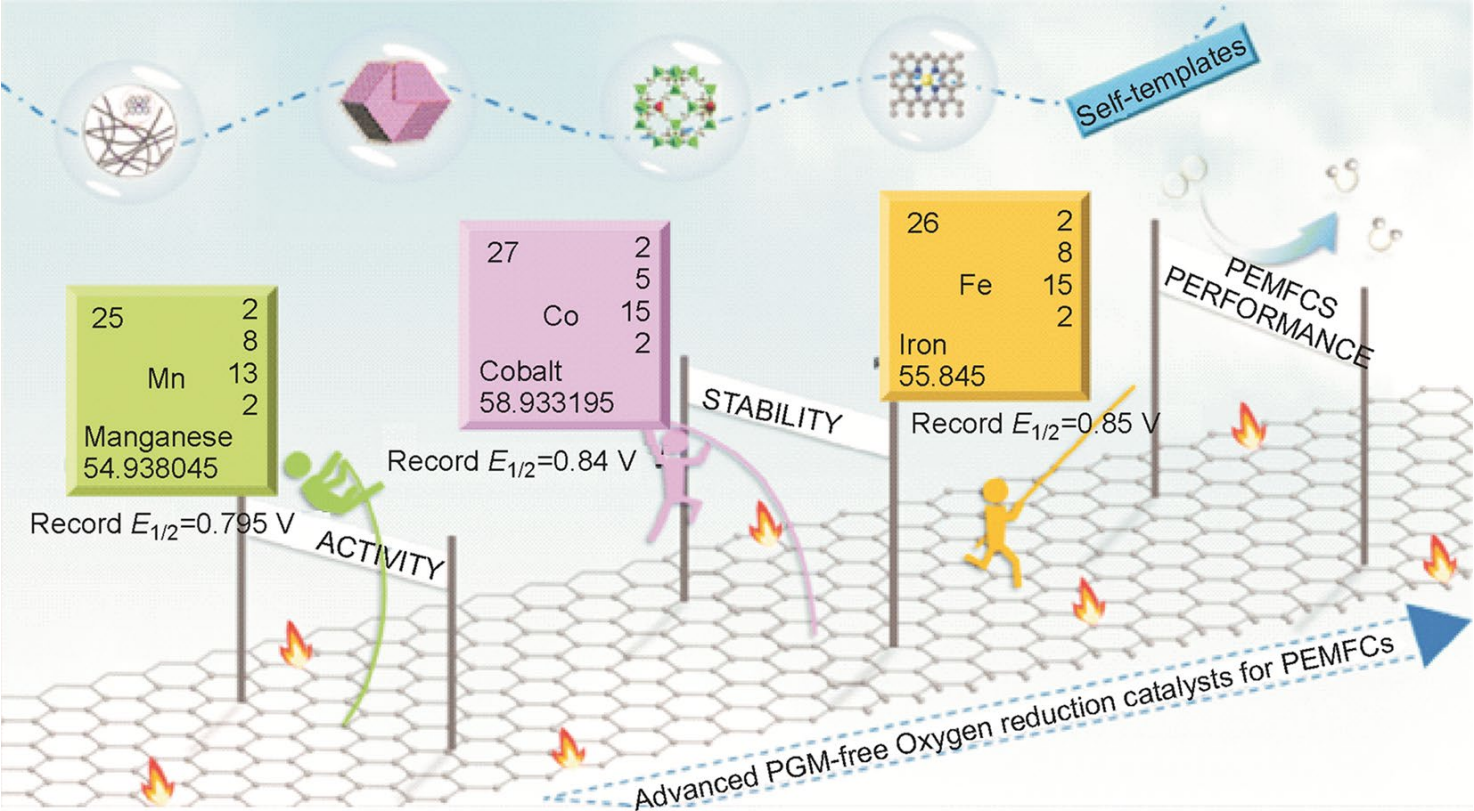
The development of M–N–C catalysts [55]

Fe–N–C catalyst is used as an accelerator for the generation of highly oxidative free radicals via the so-called Fenton reaction, which will significantly decrease the PEM fuel cell durability, so that the iron-free and PGM-free catalysts, e.g., Co–N–C and Mn–N–C catalysts, have been developed [48–54]. Since this iron-free catalyst concept is more relevant to the durability issues, it will be discussed in Sect. 4.2. It should be emphasized that even though iron-free catalysts develop fast, their comprehensive performance is, in general, still lower than Fe–N–C catalysts (Fig. 11). Great effort is needed to improve the performance of iron-free catalysts.

#### Improvement Strategies Beyond Catalysts

For an MEA with PGM-free catalysts at cathodes, the configuration is much more complicated, which involves ionomer in catalyst layers, gas diffusion layers, and bipolar plates with flow field, etc., in addition to catalysts. Although these aspects are out of the scope of this paper, it should be emphasized that the properties of these components significantly determine the MEA performance. Therefore, to improve the PEM fuel cell performance, efforts are also required on these components beyond catalysts. For example, the ionomer and its ratio to the catalyst in the catalyst layer [56], catalyst loading [57], cathode structure [58], and flow fields [59] have been reported as key factors influencing the MEA performance.

## Recent Progress of Highly Durable/Stable PGM-Free Catalysts

Compared with the PGM catalysts, the durability/stability of MEA made with PGM-free catalysts is usually worse. As discussed in Sect. 2.2, the poor durability/stability of PGM-free catalysts becomes one of the greatest challenges for PEM fuel cell development [12, 13]. In the past years, achievements in terms of understanding degradation mechanisms and improving durability/stability have been made for PGM-free catalysts for PEM fuel cells.

### Understanding Degradation Mechanisms

The degradation mechanisms of PGM-free MEAs have been investigated for decades. Overall, MEA degradation is complicated and involves several key factors such as membrane degradation, ionomer degradation, contaminant poisoning, as well as PGM-free catalyst degradation. This work primarily focuses on the degradation mechanisms of PGM-free M–N–C catalysts.

Several possible degradation mechanisms for PGM-free catalysts have been proposed such as the dissolution of TM, carbon corrosion, poisoning of active sites, and micropore flooding. [12, 14, 60]. So far, the demetallation of MN*x* moieties and carbon corrosion are the most investigated degradation mechanisms for PGM-free catalysts under PEM fuel cell conditions.

**Fig. 12:**
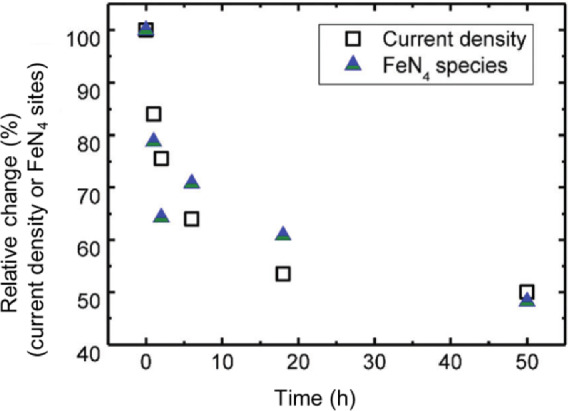
The relative changes vs. time for the number of FeN_x_ sites in MEA [21]

The Fe–N–C is the most investigated catalyst in degradation mechanisms. The FeN*x* moieties in Fe–N–C catalysts are stable in thermodynamics [22, 23]. However, the Fe dissolution was observed by in situ ICP/MS [5]. The loss of Fe in catalysts was also confirmed using the real-world MEA during stability tests [21]. As shown in Fig. 12, under the current density provided by the same MEA in the H_2_/air fuel cells at 0.6 V and 80 °C, the degradation in the current density of an MEA in PEM fuel cells is remarkably related to the loss of FeN*x* moieties, which was confirmed by the Mössbauer spectra and neutron activation analysis. Therefore, a specific demetallation mechanism was proposed to explain such a degradation phenomenon [21]. The available FeN*x* moieties locate in the open porous structures where the water comes in and out fast during the operation. Under this condition, the Le Chatelier equilibrium is broken and FeN*x* moieties are not stable anymore—the Fe ions start to lose, i.e., demetallation.

On the other hands, the majority of Fe–N–C catalysts are carbon. The carbon materials are not durable/stable under PEM fuel cell conditions [61]. In a project, the carbon dioxide emission was successfully in-situ detected at 0.3 V during the PEM fuel cell tests [62, 63]. In Fig. 5, it can be seen that with 1 k cycles, the concentration polarization becomes smaller (low cell voltage range) for the cycled MEA compared with the beginning of life. This might be related to the earlier carbon corrosion and new porous structure generation during AST cycling, which provides additional channels for mass transfer. Further, the deep carbon oxidation and corrosion (e.g., more cycles) will be remarkably harmful to the fuel cell durability. Once the carbon matrix is severely damaged, the hosted FeN*x* active moieties will be easily leached out; meanwhile, the porous structure collapse will be triggered. Particularly, in the presence of free radicals, which are generated by the iron ions and peroxide via Fenton reactions, the oxidation and corrosion would be more severe because of the highly oxidative environments. Recently, it was reported that the Co–N–C catalyst also suffers from catalyst oxidation and demetallation, leading to performance decay [54].

The reasonable models will be helpful to describe the degradation mechanisms. So far, two mathematical models have been proposed. One is the double exponential decay model proposed by the INRS team [20, 21]:

1 $$J = J_{{0,{\text{fast}}}} \exp {-}\left( {k_{{{\text{fast}}}} t} \right) + J_{{\text{0,slow}}} \exp {-}\left( {k_{{{\text{slow}}}} t} \right)$$

where *J*_0_ is the initial current contribution, and *k* is a half-life relevant rate constant of performance degradation. In this model, two sets of parameters related to exponential decay are involved to describe the two-phase decay phenomena, i.e., a fast decay (its main contribution is located at the first few tens of hours, depending on the fuel cell test conditions, for example, the first 15 h in Fig. 6) and slow decay of the current density with time. So far, the INRS team has been able to interpret the cause of the fast decay and to attribute it to the demetallation of FeN_4_ catalytic sites located in the micropores of NC_Ar + NH_3_. This requires the determination of the stability constant of FeN_4_ catalytic sites and also the use of Le Chatelier principle, since any free Fe ion that would be in equilibrium in a stagnant acid medium with its parent FeN_4_ site, would be carried away, in a working fuel cell, from its hosted micropore by the flux of water running through it. Regarding the slow decay, more efforts are still needed to identify the real reasons, which might be the demetallation of other FeN_4_ sites or if another cause, like a chemical attack by H_2_O_2_ (produced electrochemically by ORR and eventually followed by the attack of the catalyst by HO radicals generated by the Fenton reaction). On the other hands, another model is proposed by Los Alamos group [64], which is autocatalytic degradation mechanism:

2 $$J_{{k,{\text{normal}}}} = 1/\left( {1 + t/t_{0} } \right) \,$$

where *J* is the normalized current density, and *t*_0_ is an adjustable parameter. This model involves only one parameter, and the authors proposed the possible decay reason that considers H_2_O_2_ and the possible Fenton reaction as the main cause of the instability of Fe-based catalysts. Notably, this model only suits for capturing the decrease in current density in the kinetic region. That is, a high fuel cell voltage has to be intentionally selected (e.g., 0.84 V proposed by the authors in their publication, equivalent to the high potential of the cathode operation) to assure that the measured changes in current density are due to kinetic losses.

Actually, the different reasons for performance decay may be coupled, so that the degradation mechanism is complicated. Although the above-mentioned two models can describe the fuel cell degradation data well under certain conditions. A great effort is still needed for the degradation models, in terms of accuracy, universality, and predictability.

### Emerging Concepts in Improving the Durability/Stability of PGM-Free Catalysts

Similar to the performance part, some novel concepts have been proposed to address the degradation problem of PGM-free catalysts. In the following parts, the advanced Fe–N–C and catalytic materials beyond Fe–N–C will be demonstrated.

#### Advanced Fe–N–C Catalysts

**Fig. 13:**
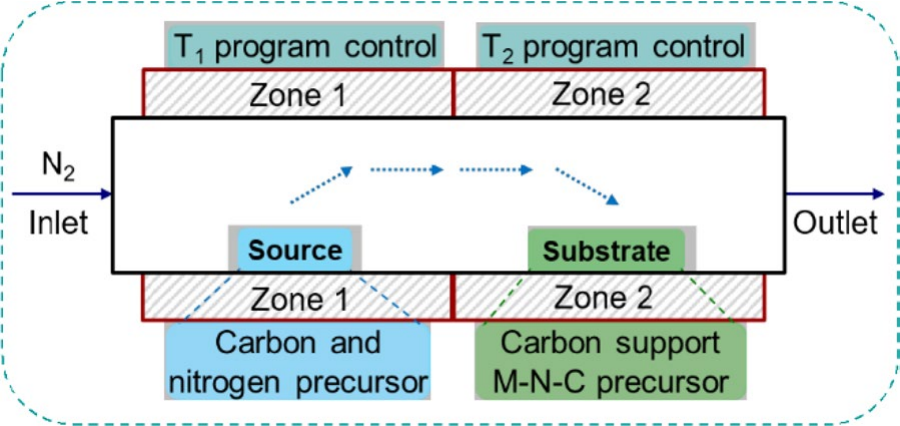
The schematic illustration for the system to synthesize the “dual-zone” catalyst [6]

To strengthen the Fe–N–C catalysts, the atomically dispersed FeN*x* moieties are helpful because each Fe atom is bonded by N atoms. Therefore, the strategies to generate well-defined atomically dispersed Fe–N–C catalysts are promising. Further, the carbon corrosion should be suppressed in Fe–N–C catalysts to improve the durability/stability. In this regard, the carbon matrix with a high degree of graphitization is a practical direction. To achieve these goals, precisely controllable synthetic methods are required. Recently, a pyrolysis method using two neighboring heat zones, whose temperatures can be precisely controlled separately, was proposed (Fig. 13) [6]. The carbon and nitrogen precursors in the 1st zone are vapored and transferred to the 2nd zone, depositing and modifying the carbon support Fe–N–C precursor. The additional carbon and nitrogen precursors from Zone 1 are supposed to help in generating a highly graphitized structure and compensating for the nitrogen loss in Zone 2. The generated Fe–N–C catalyst has a more robust carbon structure. The degradation curves of H_2_/air PEM fuel cells using this “dual-zone” catalyst demonstrate significantly improved stability. Particularly at 0.7 V, 70% of initial performance remains stable after 600-h tests. Even though this milestone is still far from the target, i.e., 5000–8000 h.

As mentioned in Sect. 4.1, the generated radicals caused by hydrogen peroxide intermediate could be a problem for durability. To address this problem, the radical scavenger could be applied and form the composite Fe–N–C/scavenger catalysts. This concept using radical scavengers, e.g., oxides, has been studied and the resulted catalysts present enhanced durability [65].

#### Beyond Fe–N–C Catalysts

**Fig. 14:**
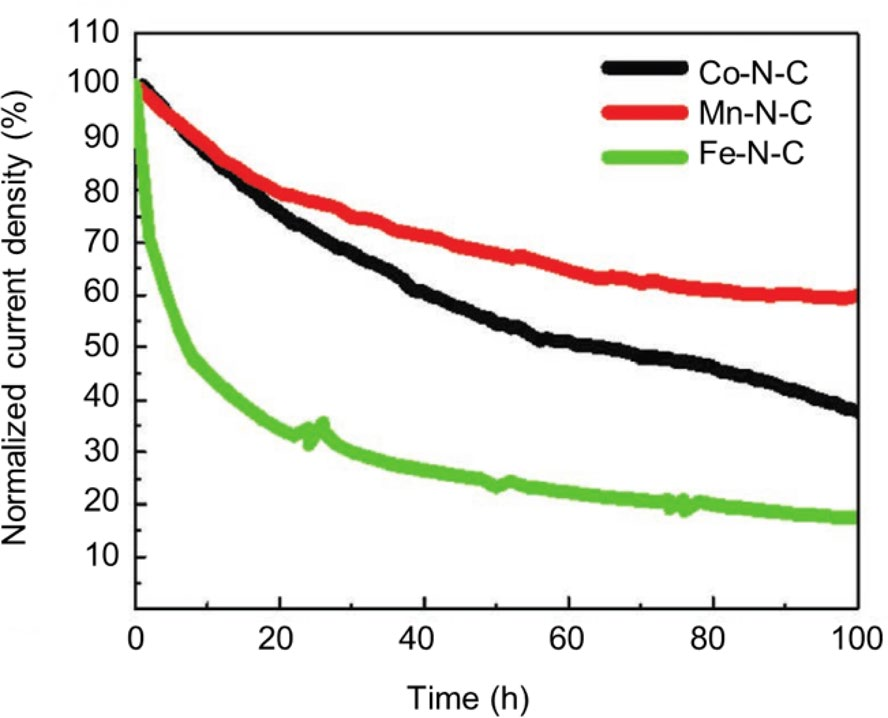
Comparison of stability at the cell voltage of 0.7 V for typical Co-N-C, Mn-N-C and Fe-N-C catalysts in H_2_/air PEM fuel cells [12]

Since Fe–N–C has unsatisfied durability/stability in PEM fuel cells, which could be due to the Fenton reaction, the iron-free catalysts have been proposed to avoid using Fe and to increase the stability. Figure 14 provides a comparison among typical Co–N–C [66], Mn-N–C [50] and Fe–N–C [21] catalysts in terms of the current stability at the cell voltage of 0.7 V of PEM fuel cells in air. It can be seen that the Co–N–C and Mn–N–C have better-normalized stability than the state-of-the-art Fe–N–C catalyst. This supports that the iron-free and PGM-free catalysts are promising in improving the fuel cell durability. However, again, it should be emphasized that the performance of these two kinds of iron-free catalysts still needs more efforts, as discussed in Sect. 3.2.2 and shown in Fig. 11.

Despite the above-mentioned works on carbon-based materials (i.e., M–N–C catalysts), the metal oxides without carbon as catalysts possess much better stability than carbon [24, 25]. However, the milestones in this topic are still limited, especially for the investigations using MEA in PEM fuel cells [67]. This might be due to the low conductivity and low surface area of metal oxides.

#### Improvement Strategies Beyond Catalysts

The degradation of MEA is related to other factors besides catalysts, such as the membrane, ionomer, and contaminants. The PGM-free catalysts are less sensitive to contaminants than PGM ORR catalysts [68]. Therefore, developing durable/stable membranes and ionomers is important to increase the overall fuel cell lifetime.

The structure of MEA determines durability/stability. Once the carbon materials at the cathode are oxidized and corroded, the structure collapse might take place and hinder mass transfer. The electrode with highly ordered configuration and robust channels for mass transfer will significantly improve the fuel cell durability/stability [12].

**Fig. 15:**
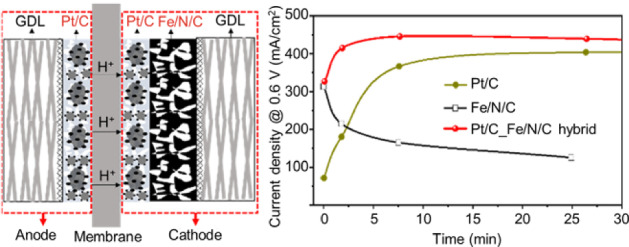
Illustration for the hybrid catalyst layer and stability curves of MEAs using Pt/C, Fe-N-C and hybrid catalyst layers [71]

Although this paper focuses on the PGM-free catalysts, it is necessary to mention the concept of hybrid PGM and PGM-free catalysts. The PGM-free catalysts still have unsatisfied stability compared with PGM catalysts, while the low-loading PGM catalysts need a long time (more than 10 h) for activation in MEA. Besides, PGM materials have a high catalytic capability to dissociate the harmful peroxide intermediate [69]. Therefore, it would be helpful to combine these two types of materials. The synergistic interaction between PGM and PGM-free sites demonstrates promises. In Ref. [70], the optimized hybrid PtCo/Co–N–C catalyst showed 64% of initial performance after 30 k AST cycles (referring to the protocol and targets for PGM catalysts [12]). Recently, the INRS team proposed a concept using hybrid Pt/C and Fe–N–C catalyst layers [71]. As shown in Fig. 15, the thin Pt/C catalyst layer (using a very low loading of 0.035 mg_Pt_/cm^2^, which is only ~ 30% of the target, 0.1 mg_Pt_/cm^2^) is fabricated between the membrane and Fe–N–C catalyst layer, generating a hybrid catalyst layer. The mass activity of this MEA reaches 0.22 A · mg_Pt_^−1^ at 0.9 V_iR-free,_ which is half of the target (0.44 A · mg_Pt_^−1^ at 0.9 V_iR-free_). The activation time is shortened to only a few hours for the hybrid catalyst layer versus over 10 h for the Pt/C catalyst layer with the same loading. Particularly, the evolution of current density at 0.6 V versus time using different catalyst layers demonstrates that the hybrid catalyst layer presents higher performance than both Pt/C and Fe–N–C and has comparable/improved stability compared with the commercial Pt/C catalyst layer (Fig. 15).

## Discussion

As presented above, the intrinsic activity of PGM-free catalysts needs further improvement. The current density at 0.9 VIR-free of the most advanced PGM-free catalysts approaches 35 mA/cm^2^, which is still lower than but approaching the target, i.e., 44 mA/cm^2^. A deep understanding of active moieties is necessary to direct the rational design and synthesis of desired catalysts. For example, the detailed configuration, coordination and structure of FeN_x_ moieties should be uncovered. To get insights into these, the advanced characterizations, especially the in-situ/operando technologies are helpful; the AI-based tools, such as machine learning, theoretical calculation and simulation, are promising in assisting the investigations of real active moieties and even predicting high-performance active moieties. Atomically dispersed FeN_x_ moieties deliver state-of-the-art performance. To further improve Fe-N-C catalysts, more exposed active sites, i.e., high active site density, are needed; meanwhile, novel active moieties such as binary FeMN_*x*_ and multiple-atom Fe_x_N_*y*_ moieties might be promising. Accordingly, developed material science and technology are needed to implement the controllable synthesis of the desired active moieties. Besides, one aspect that was not regarded as important as the active moieties is the porous structure in catalysts. Template and selftemplate methods should be developed to control the optimal porous structure of PGM-free catalysts.

On the other hand, the degradation issue seems much more severe compared to the performance problem for PGM-free catalysts. The most state-of-the-art lifetime of MEA with PGM-free cathode is still less than 1000 hours, far inferior to the target, i.e., 5000 to 8000 hours for light-duty fuel cells. The in-situ/operando characterizations for PEM fuel cells are of great help to monitor the changes in the cathode and to reveal the underlying degradation mechanisms. Importantly, the test protocols for durability (cycling) and stability (at constant voltage and current) of PGM-free catalysts vary in different research teams and laboratories, in contrast to the widely accepted cycling protocol for PGM catalysts. Therefore, more efforts are needed to work out reasonable test protocols for degradation mechanism research. New concepts such as highly graphitized carbon support, radical scavenger, iron-free catalysts and hybrid PGM and PGM-free catalyst layers help improve fuel cell durability and stability.

For either performance or durability/stability, the study at MEA level is preferred, rather than the widely used half-cell tests, i.e., rotating disk electrode. This is because the good properties in half-cell tests usually cannot be well reproduced in the MEA, which is more relevant to the real-world fuel cells. The gaps between MEA and half-cell in terms of performance and durability/stability have not been well defined. It is still hard to precisely predict the MEA performance and durability/stability based on the data obtained by half-cell tests. In this regard, the study using MEA and even real PEM fuel cell systems (e.g., fuel cell stacks consisting of many individual cells connected in series) should be encouraged.

The PGM-free catalysts are the primary focus of this work. However, it should be noted that the PEM fuel cell involves various components and factors in addition to MEA, such as the bipolar plates, gas, heat and water management systems, control systems [59]. Integration of MEAs into the fuel cell stacks also requires tremendous effort.

## Conclusions

PEM fuel cells are a promising candidate to compete with incumbent and alternative technologies as the power source for electronic vehicles. To address the cost issue, PGM-free catalysts for cathode have become one of the most fast-growing fields in the past two decades. In industrial fields, advanced companies, such as Ballard Power Systems, have achieved milestones in commercializing PEM fuel cells using PGM-free catalysts. On the other hand, various targets have been proposed in terms of performance and durability for the system and the components of fuel cells to push the development of this technique and accelerate the wide deployments. Significant achievements have been made to improve the PGM-free catalysts in PEM fuel cells. Currently, the initial performance has gradually approached the target; however, the durability is still far behind the requirement.

In the future, the intrinsic activity of PGM-free catalysts is expected to further improve, approach and even surpass 44 mA/cm^2^. This requires more insights into revealing the structure property relationships of active moieties and advanced synthetic strategies. Besides, the severe performance degradation significantly hinders the deployment of PGM-free catalysts. More efforts are needed in understanding the degradation mechanisms and exploring new concepts to improve fuel cell durability and stability. It is emphasized that future research is encouraged to be carried out at MEA level and even in real PEM fuel cell systems, instead of three-electrode level.

## Abbreviations

EV: Electric vehicle
MEA: Membrane electrolyte assembly
ORR: Oxygen reduction reaction
PEM: Proton exchange membrane
PGM: Platinum group metal

## Declaration

The copyrights of Figs. 1, 7, and 8 are attributed to Elsevier, that of Figs. 2, 4, 5, 9, 10, 13 are attributed to the US DOE, Fig. 3 to Wiley, Fig. 6 to The Royal Society of Chemistry, Fig. 11 to Springer Nature, Fig. 12 to the Royal Society of Chemistry, Fig. 14 to Wiley, and Fig. 15 to the American Chemical Society
